# Electrochemical Magnetization Switching and Energy Storage in Manganese Oxide filled Carbon Nanotubes

**DOI:** 10.1038/s41598-017-14014-7

**Published:** 2017-10-19

**Authors:** Alexander Ottmann, Maik Scholz, Marcel Haft, Elisa Thauer, Philip Schneider, Markus Gellesch, Christian Nowka, Sabine Wurmehl, Silke Hampel, Rüdiger Klingeler

**Affiliations:** 1 Kirchhoff Institute of Physics, Heidelberg University, 69120 Heidelberg, Germany; 2 Leibniz Institute for Solid State and Materials Research (IFW) Dresden, 01069 Dresden, Germany; 3 Institute for Physics of Solids, Technical University of Dresden, 01062 Dresden, Germany; 4 Centre for Advanced Materials (CAM), Heidelberg University, 69120 Heidelberg, Germany

## Abstract

The ferrimagnetic and high-capacity electrode material Mn_3_O_4_ is encapsulated inside multi-walled carbon nanotubes (CNT). We show that the rigid hollow cavities of the CNT enforce size-controlled nanoparticles which are electrochemically active inside the CNT. The ferrimagnetic Mn_3_O_4_ filling is switched by electrochemical conversion reaction to antiferromagnetic MnO. The conversion reaction is further exploited for electrochemical energy storage. Our studies confirm that the theoretical reversible capacity of the Mn_3_O_4_ filling is fully accessible. Upon reversible cycling, the Mn_3_O_4_@CNT nanocomposite reaches a maximum discharge capacity of 461 mA h g^−1^ at 100 mA g^−1^ with a capacity retention of 90% after 50 cycles. We attribute the good cycling stability to the hybrid nature of the nanocomposite: (1) Carbon encasements ensure electrical contact to the active material by forming a stable conductive network which is unaffected by potential cracks of the encapsulate. (2) The CNT shells resist strong volume changes of the encapsulate in response to electrochemical cycling, which in conventional (i.e., non-nanocomposite) Mn_3_O_4_ hinders the application in energy storage devices. Our results demonstrate that Mn_3_O_4_ nanostructures can be successfully grown inside CNT and the resulting nanocomposite can be reversibly converted and exploited for lithium-ion batteries.

## Introduction

Downsizing well-established materials to the nanoscale is a key route towards novel functionalities, in particular if different functionalities are merged in hybrid nanomaterials. One example are manganese oxides which show a relatively low electromotive force, high natural abundance, and environmental benignity and hence are considered as one of the best candidates for an anode material in lithium-ion batteries (LIB)^1^. Manganese features a plethora of stable oxidation states and thus forms various oxides, such as Mn(II)O, Mn_2_(III)O_3,_ Mn_3_(II,III)O_4_, and Mn(IV)O_2_. A promising theoretical specific capacity of 937 mA h g^−1^ in case of Mn_3_O_4_, which is nearly three times higher than that of graphite (372 mA h g^−1^)^2^, has raised considerable interest in Mn_3_O_4_ as anode material^3–6^. However, the strong fading of the electrochemical capacity due to fractionation, resulting from pronounced volume changes associated with the conversion reaction, as well as low electric conductivity seriously hinder its applicability in secondary batteries^1,7^. Nanosizing promises enhanced capability to accommodate strain, which is induced by electrochemical cycling, and may reduce kinetic limitations of the macroscopic counterparts of electrode materials^8,9^. Consequently, various manganese oxide/carbon based hybrid nanomaterials have been reported to at least partly solve these issues. These hybrid materials show enhanced electrochemical properties, such as high specific capacities and good cycling stability^10–14^. Carbon nanotube (CNT) based composites include MnO/CNT mixtures^15^ as well as the exohedral functionalization of CNT by Mn_3_O_4_^2^. So far, however, neither the synthesis of Mn_3_O_4_-filled CNT nor electrochemical studies on this material have been reported, as previous studies solely addressed manganese oxide particles located on the outer surface of the nanotubes^2,11,16,17^. Owing to their excellent conductivity, chemical stability, and very high mechanical strength, CNT as such have indeed proven to be a promising choice as carbon source in hybrid nanomaterials^18^. However, in conventional approaches using exohedrally functionalized CNT the synthesis of uniformly sized and shape-controlled nanoparticles of different manganese oxides is an unsolved challenge. The approach presented here applies the wet chemical synthesis of crystalline Mn_3_O_4_ nanoparticles inside the inner hollow cavities of multi-walled carbon nanotubes (i.e., Mn_3_O_4_@CNT) via a solution-based approach.

## Results and Discussion

**Figure 1:**
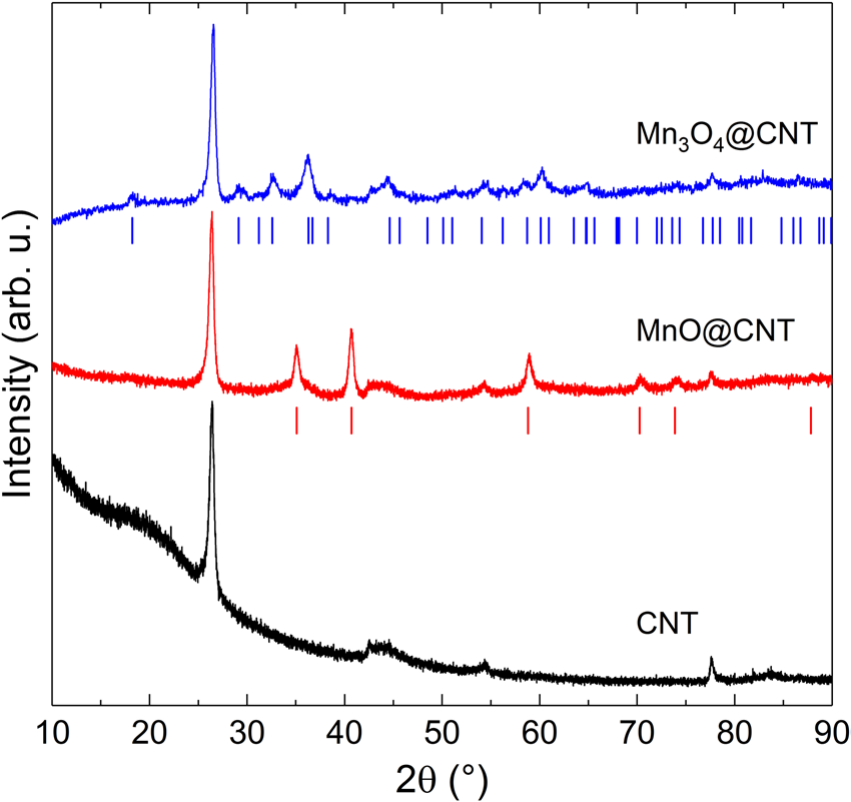
XRD patterns of Mn_3_O_4_@CNT, MnO@CNT, and pure CNT. Vertical lines show the Bragg positions of Mn_3_O_4_ (space group I4_1_/amd)^22^ and MnO (space group Fm $\bar{3}\,$m)^21^.

Mn_3_O_4_@CNT hybrid nanocomposites are obtained after filling CNT with a manganese salt solution and a subsequent reducing step (cf. Refs^19,20^). With a reduction profile of 4 h at 500 °C under Ar/H_2_ flow, homogeneously MnO-filled CNT (MnO@CNT) are synthesized. The XRD-pattern of MnO@CNT in comparison with reference Bragg peak positions (ICSD #162039^21^) confirms the presence of phase-pure MnO with space group Fm$\bar{3}$m (Fig. 1). Additional Bragg reflections, for instance at 2θ = 26.4°, 54.3°, and 77.7° originate from the CNT template. A subsequent temperature treatment of MnO@CNT in Ar at 350 °C for 6 h yields the complete conversion into Mn_3_O_4_@CNT, as confirmed by the XRD pattern in Fig. 1, which is characteristic for the tetragonal Mn_3_O_4_ phase with space group I4_1_/amd (ICSD #68174^22^). Again, all additional reflections are ascribed to the CNT. The XRD data hence suggests the complete oxidation of MnO to Mn_3_O_4_ upon thermal treatment. Note, that all patterns show relatively broad Bragg reflections, typical for the small size of the MnO and Mn_3_O_4_ nanocrystals.

**Figure 2:**
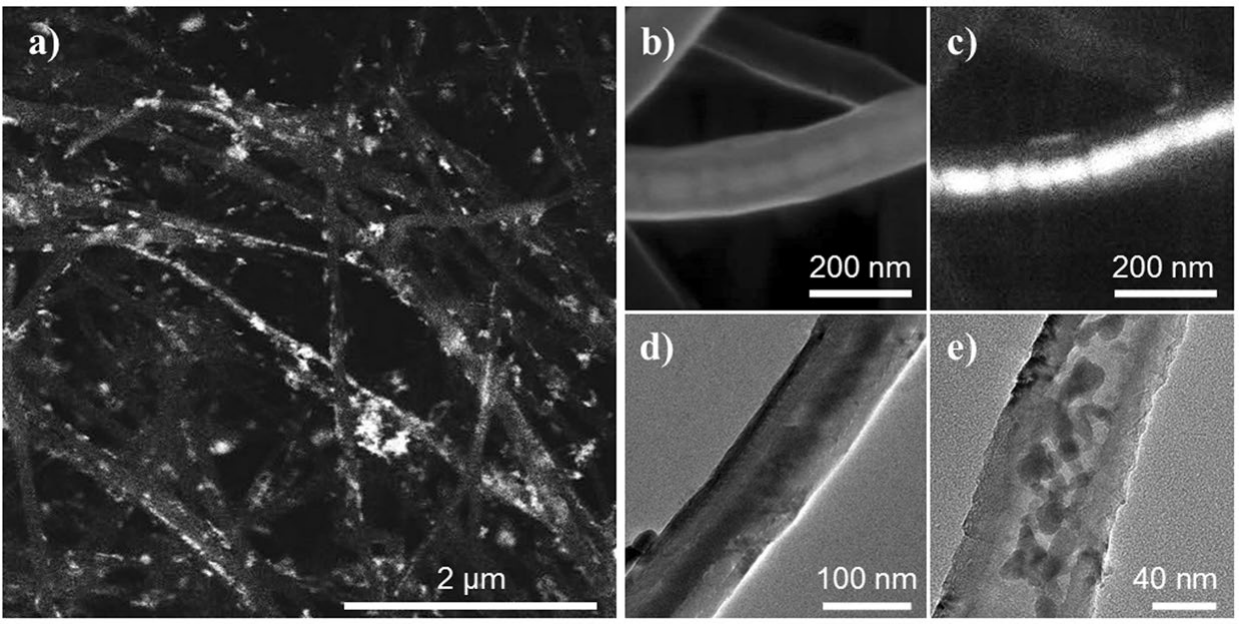
(**a**) Overview SEM image of MnO@CNT (BSE mode); (**b**) SEM image of an individual Mn_3_O_4_@CNT (SE mode); (**c**) corresponding BSE mode image of the individual Mn_3_O_4_@CNT; (**d**,**e**) TEM images of different individual Mn_3_O_4_@CNT.

Exemplary SEM and TEM images in Fig. 2 show the morphology and microstructure of both, MnO- and Mn_3_O_4_-filled CNT. In both materials, manganese oxide nanoparticles are located mainly inside the CNT. From the TGA data (Fig. S1 of the Supplementary Material) we infer a total amount of 29.5 ± 1.0 wt% Mn_3_O_4_ in Mn_3_O_4_@CNT. A more detailed inspection of the TGA graphs shows a slight increase of the sample weight before it strongly decreases. The latter signals the oxidation of the CNT as confirmed by a comparison to the curve of pristine CNT. The observed slight mass increase is in good accordance to the literature where it is attributed to the oxidation of the particles on the outside of the CNT^20^. These exohedral particles are oxidized first because they lack the protection by the CNT. Upon further heating, the CNT as well as the encapsulated material are oxidized. Differences between the curves of pristine CNT and Mn_3_O_4_@CNT are due to the chemical filling procedure which yields more defects at the surface of the CNT. In addition, the higher reactivity of the filling material itself promotes the oxidation of the CNT. To summarize, nearly the entire 30 wt% of Mn_3_O_4_ is inside the CNT while a small amount of at most 5 wt% is exohedrally attached. The encapsulated, rather spherical nanoparticles are arranged in pearl necklace-like structures (Fig. 2b and c) with lengths of more than several hundred nanometers. For both, MnO@CNT and Mn_3_O_4_@CNT, the average diameter of the encapsulated particles amounts to 15 ± 7 nm (30 particles of each oxide were surveyed in TEM), which is smaller than the size-limiting inner diameter of the utilized CNT (~35 nm). The broad size distribution of the oxidic particles inside the CNT reflects that, in addition to the particles featuring pearl necklace-like structures, MnO- and Mn_3_O_4_@CNT exhibit smaller nanoparticles (Fig. 2d and e). While comparing the images of MnO- and Mn_3_O_4_@CNT samples, no apparent differences regarding the shape of the filling particles are observed. The results hence show that our method, involving prefabricated CNT as a template, results in the formation of carbon-shielded crystalline nanoparticles with a well-defined diameter distribution, which is determined by the inner diameter of the CNT.

**Figure 3:**
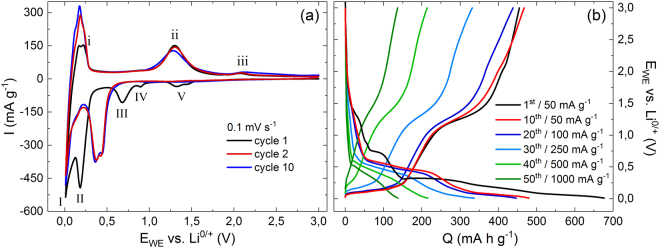
(**a**) Cyclic voltammogram of Mn_3_O_4_@CNT at 0.1 mV s^−1^. (b) Voltage profiles of the rate capability test with specific charge/discharge currents from 50 to 1000 mA g^−1^.

The electrochemical performance of the Mn_3_O_4_@CNT composite is studied by means of cyclic voltammetry and galvanostatic cycling with potential limitation (GCPL), both in the range of 0.01–3.0 V vs. Li/Li^+^. Figure 3a shows the 1^st^, 2^nd^, and 10^th^ cycle of a cyclic voltammogram (CV), recorded at a scan rate of 0.1 mV s^−1^. During the initial cycle, starting with the cathodic scan, five distinct reduction peaks (*I-V*) and three oxidation peaks (*i-iii*) are observed. The redox pair *I/i* around 0.1 V and the irreversible reduction peak *III* at 0.7 V can be attributed to processes related to multi-walled CNT, as supported by the CV of unfilled CNT which shows the same features (see Fig. S2). The irreversible reduction *III* corresponds to the formation of a passivating solid electrolyte interphase (SEI) on the surface of the CNT^23^. The pronounced redox pair *I/i* indicates intercalation and deintercalation of Li^+^-ions between the graphitic layers of the CNT^24,25^. In this regard, the slight splitting of the oxidation peak *i* resembles the staging phenomenon reported for graphite electrodes^26^. The observed increase of this peak intensity (*i*) during cycling is also found in the CV of the unfilled CNT (Fig. S2).

All other observed redox features can be associated with the electrochemical reaction mechanism of Mn_3_O_4_. Even though partially overlapping^4^, the following reaction steps are involved^27,28^:

A $${{\rm{M}}{\rm{n}}}_{3}(1/3\cdot {\rm{I}}{\rm{I}},\,2/3\cdot {\rm{I}}{\rm{I}}{\rm{I}}){{\rm{O}}}_{4}+{{\rm{L}}{\rm{i}}}^{+}+{e}^{-}\,\to \,{{\rm{L}}{\rm{i}}{\rm{M}}{\rm{n}}}_{3}(2/3\cdot {\rm{I}}{\rm{I}},\,1/3\cdot {\rm{I}}{\rm{I}}{\rm{I}}){{\rm{O}}}_{4}$$

B $${{\rm{LiMn}}}_{3}{{\rm{O}}}_{4}+{{\rm{Li}}}^{+}+{{\rm{e}}}^{-}\,\to \,{{\rm{Li}}}_{2}{\rm{O}}+3\cdot {\rm{Mn}}({\rm{II}}){\rm{O}}$$

C $${\rm{Mn}}({\rm{II}}){\rm{O}}+2\cdot {{\rm{Li}}}^{+}+2\cdot {{\rm{e}}}^{-}\leftrightarrow {{\rm{Li}}}_{2}{\rm{O}}+{\rm{Mn}}(0)$$

During the first cathodic scan (Fig. 3a), the intercalation of Li^+^ into Mn_3_O_4_ (A) is reflected by the reduction peak *V* at 1.3 V vs. Li/Li^+^. The adjacent shoulder around 1.45 V may be associated with the reduction of small amounts of amorphous manganese oxides with a higher Mn oxidation state than that in Mn(II,III)_3_O_4_^4^, e.g., Mn(II,IV)_5_O_8_^29^. Proceeding to lower potentials, peak *IV* at 0.9 V is associated with the reduction of LiMn_3_O_4_ to MnO (B); and the pronounced reduction peak *II* corresponds to the conversion of MnO to metallic Mn (C). The associated oxidation from Mn back to MnO is reflected by peak *ii* at 1.3 V during the first anodic scan. The additional oxidative feature *iii* around 2.1 V might display the back-formation of Mn_3_O_4_^30,31^. Interestingly, this feature occurs in only few studies^3,12,29,31^, which might point to enhanced reaction kinetics promoted by the conductive CNT network^3^. In the second cycle, the reportedly irreversible reduction peaks *IV* and *V* have nearly vanished, while peak *II* splits into a double peak and shifts to approx. 0.4 V. The shift indicates a structural transformation due to conversion reaction (C) where disordered Mn and Li_2_O are formed accompanied by a volume expansion^4,32,33^. There are no significant changes between cycle 2 and 10, which indicates the good cycling stability of the Mn_3_O_4_@CNT nanocomposite.

**Figure 4:**
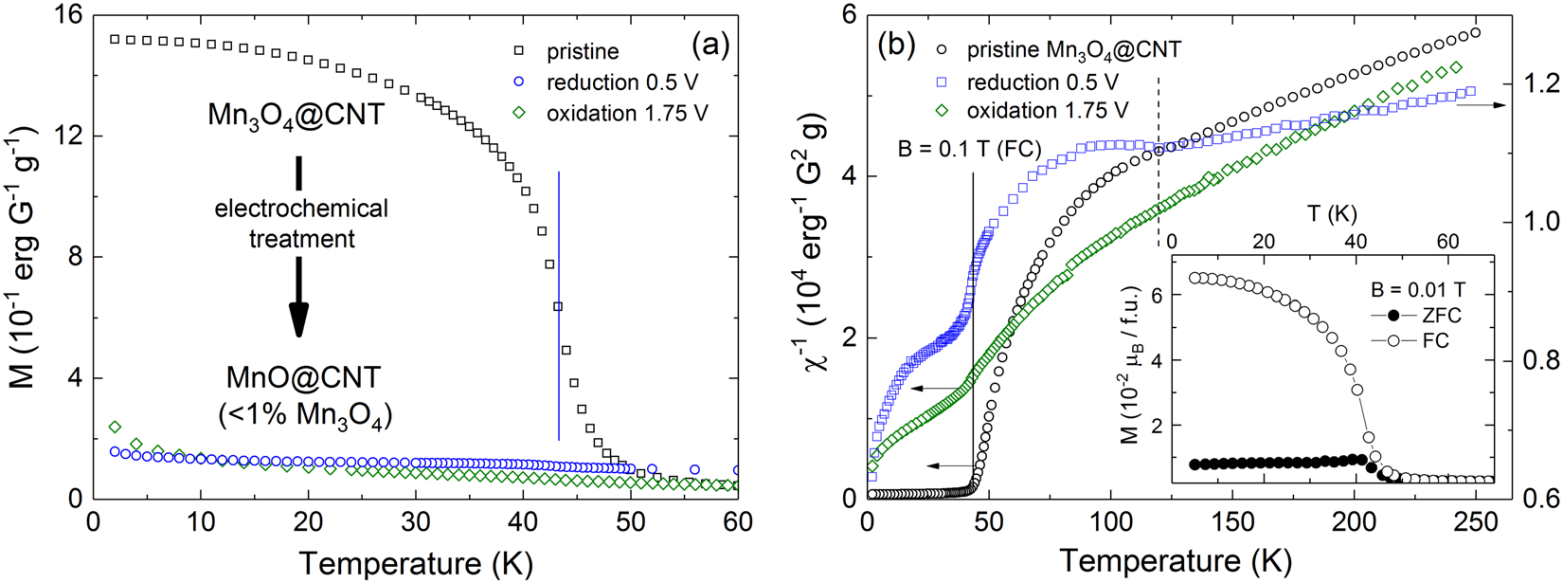
Magnetization (**a**) and inverse magnetic susceptibility (**b**) of pristine and electrochemically cycled Mn_3_O_4_@CNT, measured at B = 0.1 T (FC); note the different ordinate scales in (**b**). The solid (dashed) vertical line indicates the ferrimagnetic (antiferromagnetic) ordering temperature in Mn_3_O_4_ (MnO). Inset (**b**): Field-cooled/zero-field-cooled magnetization of Mn_3_O_4_@CNT obtained at B = 0.01 T.

The quality and ratio of the Mn_3_O_4_@CNT filling as well as the presence of intermediate products appearing upon electrochemical cycling is also confirmed by magnetization studies, as such experiments have been reported to be an appropriate tool to confirm electrochemical conversion in transition metal oxides^34–36^. Indeed, the magnetic properties of the associated manganese oxides differ strongly so that large magnetization changes are expected. The conversion reactions (A) and (B) reflect the switching of ferrimagnetic Mn_3_O_4_ nanoparticles to antiferromagnetic MnO. The respective magnetic ordering phenomena appear at T_C_ = 42 K in Mn_3_O_4_ and T_N_ = 120 K in MnO^37–39^. Switching of the magnetic properties upon electrochemical treatment is directly confirmed by the magnetization data in Fig. 4. As shown in the inset of Fig. 4, pristine Mn_3_O_4_@CNT indeed shows a long-range magnetic order at T_C_ = 42 ± 1 K, confirming the ferrimagnetic nature of the encapsulate. Upon galvanostatic lithiation at 5 mA g^−1^ down to 0.5 V, i.e. after passing the reduction peaks *V*, *IV*, and *III* (cf. Figure 3a), the large magnetic moment vanishes when switching the ferrimagnetic filling to an antiferromagnetic one. The latter exhibits antiferromagnetic order below ~120 K as shown by the inverse magnetic susceptibility, which is depicted in Fig. 4b. This observation confirms the presence of MnO in agreement with Equation (B), i.e., the switching of the ferrimagnetic Mn_3_O_4_ encapsulate to the antiferromagnet MnO. As the magnetization measures bulk properties of the samples, the data imply that, after the lithiation, there is only about 1% remainder of pristine Mn_3_O_4_ while 99% of the material has been converted during the first half cycle. Material extracted at 1.75 V during the subsequent delithiation shows an even weaker signature of ferrimagnetism and no feature at 120 K. This finding refers to the fact that MnO appears to be amorphous after the first complete charge/discharge cycle which will suppress long-range magnetic order^4^.

**Figure 5:**
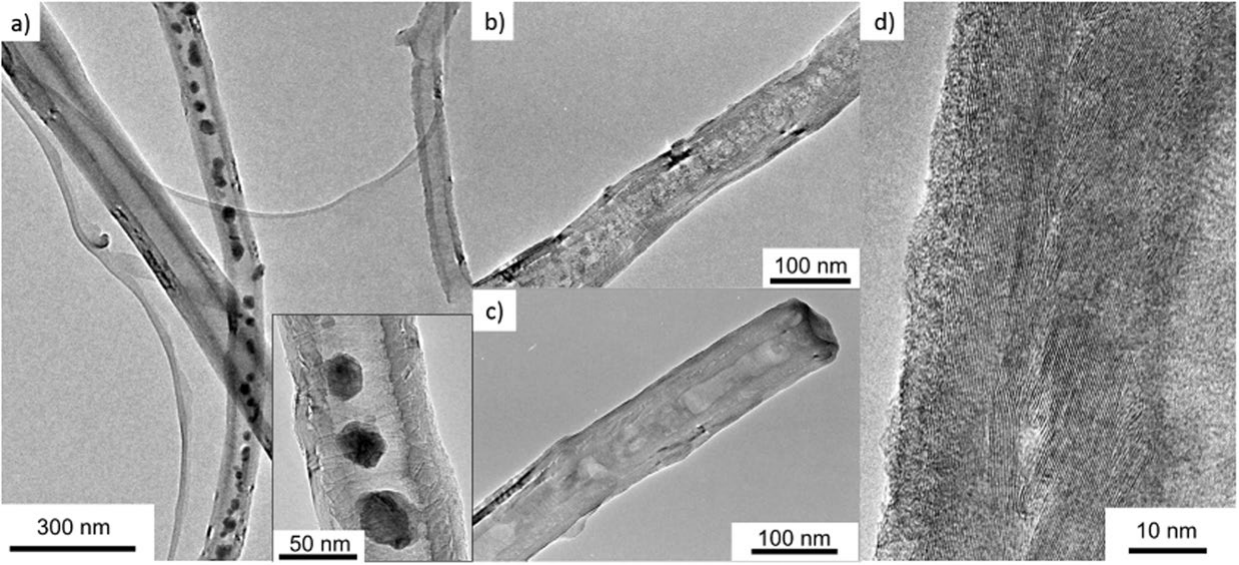
TEM images of (**a**) uncycled, (**b**) galavanostatically lithiated, and (**c**) delithiated Mn_3_O_4_@CNT. (**d**) High-resolution TEM image of a CNT shell of delithiated material after 13 cycles.

TEM images of galavanostatically lithiated (b) and delithiated (c) Mn_3_O_4_@CNT in comparison to the pristine material (a) are displayed in Fig. 5 in order to clarify the benefits of the CNT encasements upon cycling. In the case of the uncycled material, single nanoparticles with a maximum diameter limited by the inner diameter of the hollow CNT are clearly recognizable, whereas the cycled composite shows extended patches of the encapsulate. This observation agrees with the large expected volume expansion of Mn_3_O_4_ during initial lithiation, which probably yields an agglomeration of several nanoparticles inside single CNT. The volume expansion is also apparent from a lower density of the encapsulate, which can be deduced from a lower contrast to the CNT environment in the TEM image. No clear differences are observed between the lithiated (Fig. 5b) and the subsequently delithiated (Fig. 5c) material. Figure 5d shows a high-resolution image of a delithiated CNT shell after 13 charge/discharge cycles, which still displays the characteristic graphitic layers of multi-walled carbon nanotubes. Hence, the electrochemical cycling, and in particular the volume expansion of the encapsulate do not damage the structure of the CNT. Furthermore, an amorphous layer of ~5 nm thickness can be observed on top of the graphitic CNT layers, which can be attributed to the SEI (cf. Peak *III* in Fig. 3a). The TEM analysis hence shows that the CNT indeed offer a stable environment for the manganese oxide which is able to accommodate the strain due to volume expansion during electrochemical cycling and guarantees a consistent electrical contact to the active material.

The electrochemical performance of Mn_3_O_4_@CNT is further studied by means of charge and discharge profiles of a rate capability test with specific charge/discharge currents between 50 and 1000 mA g^−1^ (Fig. 3b). In the first cycle at 50 mA g^−1^, specific charge/discharge capacities of 677/455 mA h g^−1^ are reached, and all redox features which have been discussed on the basis of the CV (Fig. 3a) can be identified as plateau-like segments or kinks in the depicted voltage profiles. The redox pairs *I/i* and *II/ii* remain clearly distinguishable in the 10^th^ cycle with plateau-like features around 0.1/0.1 V and 0.5/1.3 V, respectively. Increasing the charge/discharge current to 100 and 250 mA g^−1^, respectively, does not have a significant impact on the shape of the voltage profiles, but reduces the discharge capacity to, e.g., 331 mA h g^−1^ after 30 cycles. For higher currents, the plateaus corresponding to de-/lithiation of the CNT (*i/I*) vanish, most probably due to the overpotential caused by the ohmic resistance of the cell setup. In addition, the features corresponding to the conversion reaction (C) (*II/ii*) become more sloping but stay present.

**Figure 6:**
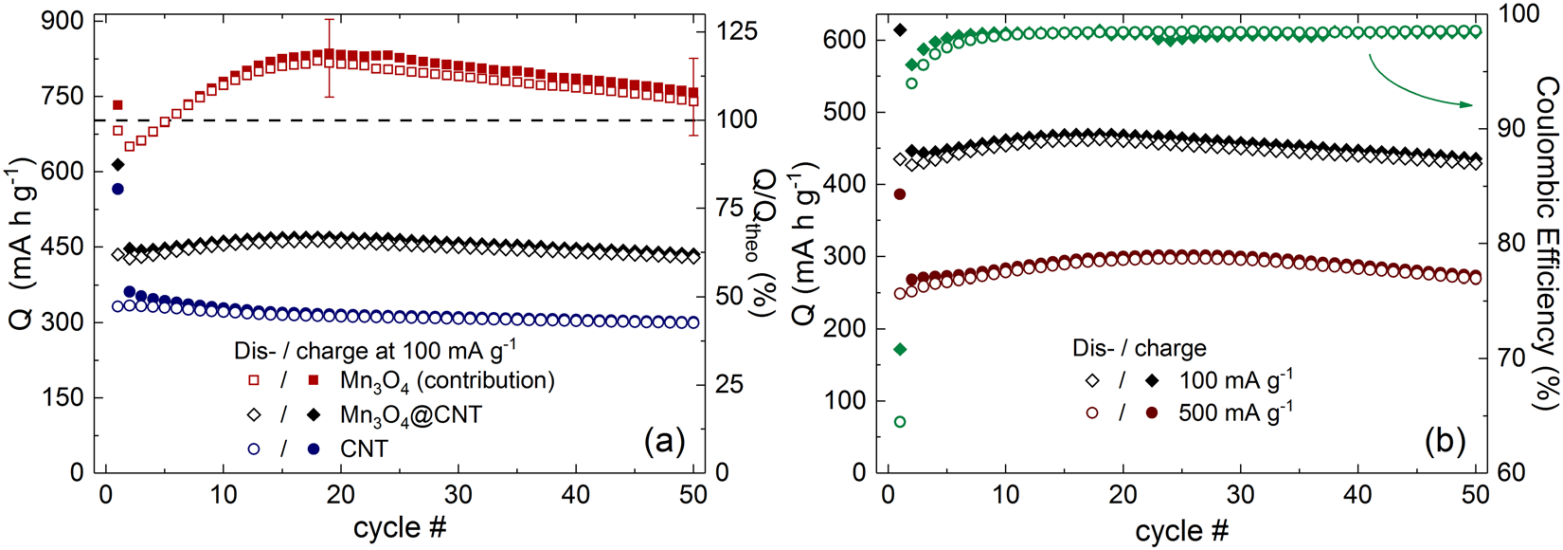
(**a**) Specific charge/discharge capacities of CNT (circles) and Mn_3_O_4_@CNT (diamonds) at 100 mA g^−1^, and the calculated contribution of the incorporated Mn_3_O_4_ (squares), based on a filling of 29.5 wt%. (**b**) Charge/discharge capacities and the corresponding coulombic efficiencies of Mn_3_O_4_@CNT at 100 mA g^−1^ (diamonds) and 500 mA g^−1^ (circles).

The progression of charge/discharge capacities of Mn_3_O_4_@CNT and pristine CNT at 100 mA g^−1^ (GCPL) are compared in Fig. 6a. Both, the composite material and the pristine CNT, show a pronounced irreversible contribution due to the SEI formation in the first half cycle with initial charge/discharge capacities of 614/435 and 566/332 mA h g^−1^, respectively. The CNT reach a maximum discharge capacity of 334 mA h g^−1^ in the 2^nd^ cycle which decreases moderately to 299 mA h g^−1^ in cycle 50. The Mn_3_O_4_@CNT composite exhibits increasing capacities for approximately 15 cycles before reaching a maximum discharge capacity of 463 mA h g^−1^ in cycle 18, of which 93% is maintained after 50 cycles (429 mA h g^−1^). Thus, the incorporation of Mn_3_O_4_ into CNT leads to more than 40% enhanced specific capacities on average as compared to unfilled CNT. The initial capacity increase was also observed in other studies on Mn_3_O_4_/CNT composites^2,11^ and could originate from Mn_3_O_4_ nanoparticles which are not properly attached to the conductive CNT network before electrochemical cycling.

In order to compare the specific capacity of the incorporated Mn_3_O_4_ (29.5 wt%) to the theoretical value associated with the reversible conversion reaction step (C), i.e. 703 mA h g^−1^, the data have been corrected for the contribution of the CNT and normalized accordingly (cf. Figure 6a (squares)). The capacity loss found in the first cycle originates from the irreversible reductions *IV* and *V* (Fig. 3a). In the further course of the GCPL, the charge/discharge capacities of the incorporated Mn_3_O_4_ first increase significantly to 829/820 mA h g^−1^ (cycle 18), and then begin to decline with a capacity retention of around 90% after 50 cycles. The contributed capacities even exceed the theoretical expectations of the conversion reaction (C) from the 6^th^ cycle on. This observation can be explained by an additional contribution due to oxidative feature *iii* (Fig. 3a), which supposedly indicates the back-formation of Mn_3_O_4_^3,31^ and corresponding reduction processes. In this context, the experimental uncertainty in the order of 8% should be considered as well, as it impedes a quantitative discussion of the excess capacities. To summarize, our analysis clearly shows that the full conversion between MnO and metallic Mn can be achieved reversibly for at least several cycles around the maximum of the contributed capacities by Mn_3_O_4_ (cf. Figure 6a). In particular, the contribution of Mn_3_O_4_ exceeding the theoretical capacity implicitly confirms that the nanoparticles inside the CNT are completely involved in the electrochemical cycling. This finding is supported by the fact that the active material inside the CNT experiences distinct structural changes, as evidenced by the TEM studies (Fig. 5).

The Mn_3_O_4_@CNT composite still performs well at an elevated current of 500 mA g^−1^ which is illustrated in comparison to the specific capacities at 100 mA g^−1^ in Fig. 6b, including the respective coulombic efficiencies. The general trends with increasing capacities after the initial cycle are similar, even though the fivefold higher charge/discharge current leads to approx. 35% reduced capacities with a maximum of 302/297 mA h g^−1^ in the 25^th^ cycle. This capacity decrease is presumably caused by kinetic limitations of the active material, and the above-mentioned overpotential due to the ohmic resistance of the cell setup. Both coulombic efficiencies reflect the strong irreversible contributions during the initial lithiation mainly due to the SEI formation with values of 71% (100 mA g^−1^) and 64% (500 mA g^−1^). Subsequently, they increase simultaneously to the specific capacities and demonstrate satisfying values between 98–99% from the 8^th^ cycle on. Those values indicate a decent cycling stability of the Mn_3_O_4_@CNT composite. Further measurements regarding the cycling stability at 100 mA g^−1^ confirm that the depicted decline stays very moderate with a capacity loss of approx. 15% per 50 cycles for a total of more than 100 cycles. The capacity losses can be attributed to different reasons: on the one hand, the small amount of Mn_3_O_4_ nanoparticles located on the outside of the CNT may upon volume expansion either detach or inhibit Li^+^ transfer from/into the CNT; on the other hand the CNT themselves do not offer perfect cycling stability (Fig. 6a).

**Figure 7:**
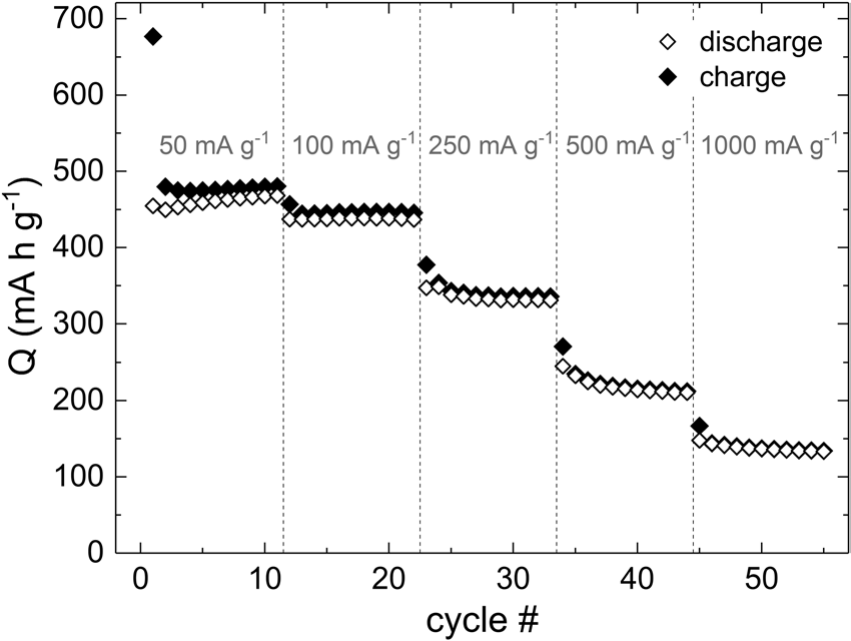
Specific charge/discharge capacities of rate capability test with 11 cycles at 50, 100, 250, 500, and 1000 mA g^−1^, respectively.

The specific capacities obtained at further charge/discharge rates are displayed in Fig. 7. While there is only a small capacity decrease from 50 to 100 mA g^−1^, further increasing the current results in pronounced capacity losses. Maximum discharge capacities of 468, 439, 349, 245, and 148 mA h g^−1^ are reached at 50, 100, 250, 500, and 1000 mA g^−1^, respectively. The values at 500 mA g^−1^ differ noticeably from the ones presented for a constant charge/discharge current (cf. Figure 6b). We attribute this observation to the fact that smaller currents, in particular 50 mA g^−1^, used in the beginning of the rate capability test (Fig. 7) lead to a more complete charge/discharge process and therefore to more pronounced degradation effects.

## Conclusions

The hybrid nanomaterial Mn_3_O_4_@CNT, produced from encapsulation of Mn_3_O_4_ nanoparticles inside multi-walled carbon nanotubes, is electrochemically active and can be switched from a ferrimagnet to an antiferromagnet by electrochemical lithiation. Furthermore, the associated conversion reaction can be exploited for electrochemical energy storage in LIB. While the rigid hollow of the CNT enforces size-controlled nanoparticles, the protective carbon shells do not only prevent degradation and large agglomerates beyond the inner hollow of individual CNT but also form a stable conductive network, electrically connecting the active material. This network is in particular unaffected by cracks of the encapsulate which usually inhibit long-term stability of bare nanosized transition metal oxide anode materials. Remarkably, we find a complete conversion of the active material upon cycling so that there is indeed full access to the whole theoretical capacity of Mn_3_O_4_ in the nanocomposite. In addition, a good capacity retention of around 90% after 50 cycles implies that transition metal oxide@CNT nanocomposites provide a successful route to new nanohybride anode materials for LIB.

## Methods

### Synthesis

The Mn_3_O_4_-filled CNT are a result of a three-step synthesis that can be described as follows: (1) filling the open CNT with a metal salt solution, (2) reducing the metal salt to MnO, and (3) oxidation of MnO to Mn_3_O_4_. For the first step, a solution of Mn(NO_3_)_2_·3H_2_O (analytical grade, Sigma Aldrich) with a concentration of 1 mol/L and CNT (Pyrograf Inc., inner diameter ca. 35 nm) were dispersed for 1.5 hours in an ultrasonic bath. This homogeneous dispersion was filtered and the tubes were dried. In the second step, the dry, filled CNT were reduced in an Ar/H_2_ flow (50 sccm min^−1^/50 sccm min^−1^) for 4 h at 500 °C. Subsequently, the material was tempered at 350 °C for 6 h in Ar flow (50 sccm min^−1^) to form Mn_3_O_4_@CNT.

### Characterization

The composite was characterized by X-ray diffraction (XRD, Stadi P (Stoe)) using Cu K_α1_ radiation (λ = 1.5406 Å), scanning electron microscopy (SEM, Nova NanoSEM 200, FEI Company) and transmission electron microscopy (TEM, Jeol JEM, 2010 F). SEM images were obtained either in the back scattered electrons (BSE) mode or in the secondary electrons (SE) mode. Measurements of the particle diameter were accomplished with the program ImageJ^40^. A SDT Q600 (TA Instruments) was used for thermogravimetric analysis (TGA). During TGA measurements the filled CNT were burned at a heating rate of 5 K min^−1^ up to 850 °C under the flow of synthetic air. Magnetic measurements of Mn_3_O_4_@CNT were performed by means of a MPMS-XL5 (Quantum Design) SQUID magnetometer with powder samples. The temperature was varied between 2 and 300 K according to zero-field-cooling (ZFC)/field-cooled-cooling (FCC) procedures at 100 Oe. Hysteresis loops were obtained at 5 and 300 K in magnetic fields of up to ± 5 T.

### Electrochemistry

The Mn_3_O_4_@CNT composite as well as pristine CNT were characterized electrochemically by means of cyclic voltammetry (CV) and galvanostatic cycling (GCPL). The measurements were performed in Swagelok-type two electrode cells on a *VMP3* potentiostat (BioLogic) at a constant temperature of 25 °C. The working electrodes were prepared by stirring the active material in a solution of polyvinylidene fluoride (PVDF, Solvay Plastics) in N-methyl-2-pyrrolidone (NMP, Sigma-Aldrich) overnight, and then evaporating most of the NMP in order to obtain a slurry which was spread on circular copper current collectors. The weight ratio of active material to PVDF amounted to 86:14 and the mass loading of the electrodes was 3–4.5 mg cm^−2^. Afterwards, the electrodes were dried at ~100 °C in vacuum (<5 mbar) overnight, mechanically pressed at 10 MPa, and then dried again. The cells were assembled in an argon atmosphere glove box (O_2_/H_2_O < 1 ppm), using a lithium metal foil counter electrode pressed on a nickel current collector, two layers of glass microfibre separator (Whatman *GF/D*), and 200 μl of a 1 mol l^−1^ solution of LiPF_6_ in 1:1 ethylene carbonate and dimethyl carbonate (Merck Electrolyte *LP30*).

### Data availability statement

The datasets generated during and/or analysed during the current study are available from the corresponding author on reasonable request.

## Electronic supplementary material

Supplementary Material S1-S3

## References

[1] Deng Y, Wan L, Xie Y (2014). Recent advances in Mn-based oxides as anode materials for lithium ion batteries. RSC Adv..

[2] Wang Z-H, Yuan L-X, Shao Q-G (2012). Mn3O4 nanocrystals anchored on multi-walled carbon nanotubes as high-performance anode materials for lithium-ion batteries. Materials Letters.

[3] Bai Z, Zhang X, Zhang Y (2014). Facile synthesis of mesoporous Mn3O4 nanorods as a promising anode material for high performance lithium-ion batteries. J. Mater. Chem. A.

[4] Lowe MA, Gao J, Abruña HD (2013). In operando X-ray studies of the conversion reaction in Mn3O4 lithium battery anodes. J. Mater. Chem. A.

[5] Li T (2015). Well-shaped Mn3O4 tetragonal bipyramids with good performance for lithium ion batteries. J. Mater. Chem. A.

[6] Li P (2010). Mn3O4 Nanocrystals: Facile Synthesis, Controlled Assembly, and Application. Chem. Mater..

[7] Wang C, Yin L, Xiang D (2012). Uniform Carbon Layer Coated Mn3O4 Nanorod Anodes with Improved Reversible Capacity and Cyclic Stability for Lithium IonBatteries. ACS Appl. Mater. Interfaces.

[8] Arico AS, Bruce P, Scrosati B (2005). Nanostructured materials for advanced energy conversion and storage devices. Nature Materials.

[9] Armand M, Tarascon J-M (2008). Building better batteries. Nature.

[10] Chen C (2014). Facile synthesis of graphene-supported mesoporous Mn3O4 nanosheets with a high-performance in Li-ion batteries. RSC Adv..

[11] Luo S (2014). Mn3O4 nanoparticles anchored on continuous carbon nanotube network as superior anodes for lithium ion batteries. J. Power Sources.

[12] Ma F, Yuan A, Xu J (2014). Nanoparticulate Mn3O4/VGCF Composite Conversion-Anode Material with Extraordinarily High Capacity and Excellent Rate Capability for Lithium IonBatteries. ACS Appl. Mater. Interfaces.

[13] Wang L (2013). Composite structure and properties of Mn3O4/graphene oxide and Mn3O4/graphene. J. Mater. Chem. A.

[14] Hou Y, Cheng Y, Hobson T (2010). Design and synthesis of hierarchical MnO2 nanospheres/carbon nanotubes/conducting polymer ternary composite for high performance electrochemical electrodes. Nano letters.

[15] Xu G-L (2012). Facile synthesis of porous MnO/C nanotubes as a high capacity anode material for lithium ion batteries. Chem. Commun..

[16] An G (2008). Low-temperature synthesis of Mn3O4 nanoparticles loaded on multi-walled carbon nanotubes and their application in electrochemical capacitors. Nanotechnology.

[17] Zhang H, Du N, Wu P (2008). Functionalization of carbon nanotubes with magnetic nanoparticles: general nonaqueous synthesis and magnetic properties. Nanotechnology.

[18] Dai H (2002). Carbon Nanotubes: Synthesis, Integration, and Properties. Acc. Chem. Res..

[19] Gellesch M (2013). *Facile Nanotube-Assisted Syn*thesis of Ternary Intermetallic Nanocrystals of the Ferromagnetic Heusler Phase Co2FeGa. Crystal Growth & Design.

[20] Haft M (2016). Tailored nanoparticles and wires of Sn, Ge and Pb inside carbon nanotubes. Carbon.

[21] Trukhanov SV, Troyanchuk IO, Bobrikov IA (2007). Crystal structure phase separation in anion-deficient La0.70Sr0.30MnO3 − δ manganite system. J. Synch. Investig..

[22] Jarosch D (1987). Crystal structure refinement and reflectance measurements of hausmannite, Mn3O4. Mineralogy and Petrology.

[23] Frackowiak E, Gautier S, Gaucher H (1999). Electrochemical storage of lithium in multiwalled carbon nanotubes. Carbon.

[24] Chew SY (2009). Flexible free-standing carbon nanotube films for model lithium-ion batteries. Carbon.

[25] Xiong Z, Yun Y, Jin H-J (2013). Applications of Carbon Nanotubes for Lithium Ion Battery Anodes. Materials.

[26] Winter M, Besenhard JO, Spahr ME (1998). Insertion Electrode Materials for Rechargeable Lithium Batteries. Adv. Mater..

[27] Fang X (2010). Electrode reactions of manganese oxides for secondary lithium batteries. Electrochemistry Communications.

[28] Zhong K (2010). MnO powder as anode active materials for lithium ion batteries. J. Power Sources.

[29] Gao J, Lowe MA, Abruña HD (2011). Spongelike Nanosized Mn3O4 as a High-Capacity Anode Material for Rechargeable Lithium Batteries. Chem. Mater..

[30] Kim S-W (2011). Electrochemical performance and *ex situ* analysis of ZnMn2O4 nanowires as anode materials for lithium rechargeable batteries. Nano Res..

[31] Li L, Guo Z, Du A (2012). Rapid microwave-assisted synthesis of Mn3O4–graphene nanocomposite and its lithium storage properties. J. Mater. Chem..

[32] Sun B, Chen Z, Kim H-S (2011). MnO/C core–shell nanorods as high capacity anode materials for lithium-ion batteries. J. Power Sources.

[33] Zhong K (2011). Investigation on porous MnO microsphere anode for lithium ion batteries. J. Power Sources.

[34] Reitz C, Leufke PM, Schneider R (2014). & Brezesinski, T. Large Magnetoresistance and Electrostatic Control of Magnetism in Ordered Mesoporous La1– xCaxMnO3 Thin Films. Chem. Mater..

[35] Zhang Q (2016). Lithium-Ion Battery Cycling for Magnetism Control. Nano letters.

[36] Wei G (2017). Reversible control of magnetization of Fe3O4 by a solid-state film lithium battery. Appl. Phys. Lett..

[37] Tyler RW (1933). The Magnetic Susceptibility of MnO as a Function of the Temperature. Physical Review.

[38] Djerdj I, Arčon D, Jagličić Z (2007). Nonaqueous Synthesis of Manganese Oxide Nanoparticles, Structural Characterization, and Magnetic Properties. J. Phys. Chem. C.

[39] Seo WS (2004). Size-Dependent Magnetic Properties of Colloidal Mn3O4 and MnO Nanoparticles. Angew. Chem. Int. Ed..

[40] Schneider CA, Rasband WS, Eliceiri KW (2012). NIH Image to ImageJ: 25 years of image analysis. Nat Meth.

