# Supplementary material for: Electrochemical Magnetization Switching and Energy Storage in Manganese Oxide filled Carbon Nanotubes
Source: Sci Rep. 2017 Oct 19;7:13625. doi: 10.1038/s41598-017-14014-7 (PMC5648762; doi:10.1038/s41598-017-14014-7)
Supplement: Supplementary file 1 — Supplementary Material S1-S3 [file 41598_2017_14014_MOESM1_ESM.pdf]

Supplementary Material:

Electrochemical Magnetization Switching and Energy Storage in Manganese Oxide filled Carbon Nanotubes

Alexander Ottmann[a,†,*], Maik Scholz[b,†], Marcel Haft[b], Elisa Thauer[a], Philip Schneider[a], Markus Gellesch[b], Christian Nowka[b], Sabine Wurmehl[b,c], Silke Hampel[b], Rüdiger Klingeler[a,d]

[a] Kirchhoff Institute of Physics, Heidelberg University; INF 227, 69120 Heidelberg, Germany

[b] Institute for Solid State and Materials Research (IFW) Dresden; 01171 Dresden, Germany

[c] Institute for Physics of Solids, Technical University of Dresden; 01062 Dresden, Germany

[d] Centre for Advanced Materials (CAM), Heidelberg University; INF 225, 69120 Heidelberg, Germany

[*] Corresponding author: Email: alex.ottmann@kip.uni-heidelberg.de,

Phone: +496221549819, Fax: +496221549869

[†] Both authors contributed equally

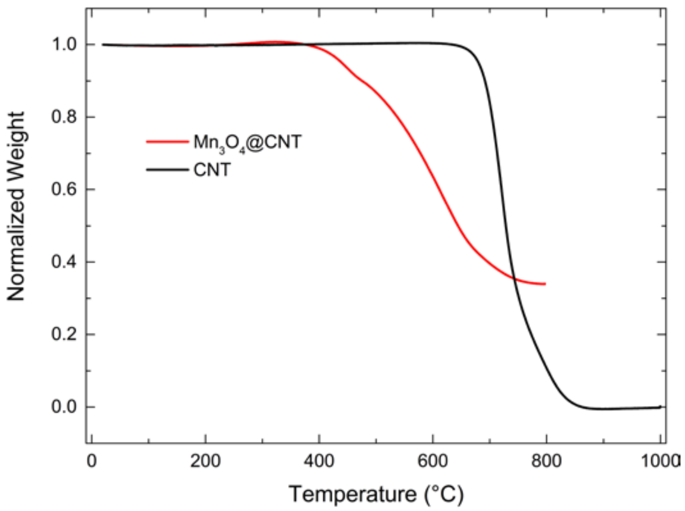

Figure S1. TGA graphs of the weight loss of Mn_3O_4@CNT and pristine CNT, respectively.

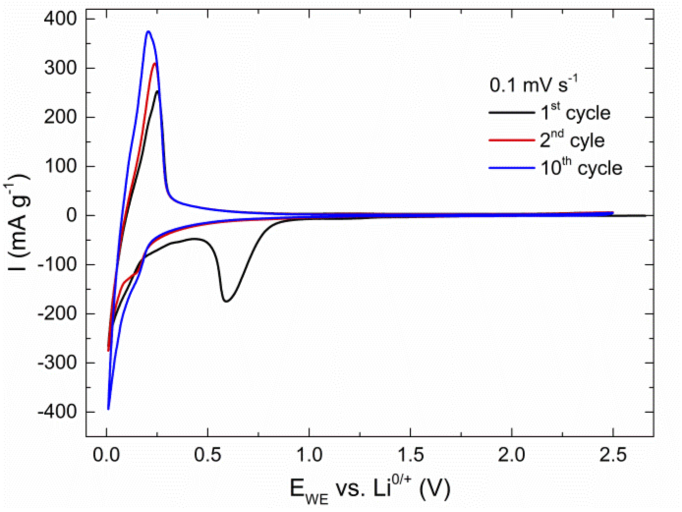

Figure S2. Cyclic voltammogram of unfilled CNT at 0.1 mV s^{-1}.

Magnetization studies

The magnetization of Mn_3O_4@CNT vs. magnetic field was measured at low temperature (Figure S1) in order to compare the coercivity H_C and the remanence M_r with the literature. Indeed, the observed values of H_C = 11.5 kOe and M_r = 0.4 μ_B/f.u. are in agreement with results reported by Bussamara et al.[1] Furthermore, it can be clearly seen that, at room temperature, i.e. far above the ferrimagnetic transition temperature, the material exhibits paramagnetic behavior.

1. R. Bussamara, Melo, Wellington W. M., J.D. Scholten, P. Migowski, G. Marin, Zapata, Maximiliano J. M., G. Machado, S.R. Teixeira, M.A. Novak, J. Dupont, Controlled synthesis of Mn3O4 nanoparticles in ionic liquids, *Dalton Trans.*, 2013, **42**, 14473-14479. DOI: 10.1039/c3dt32348j.

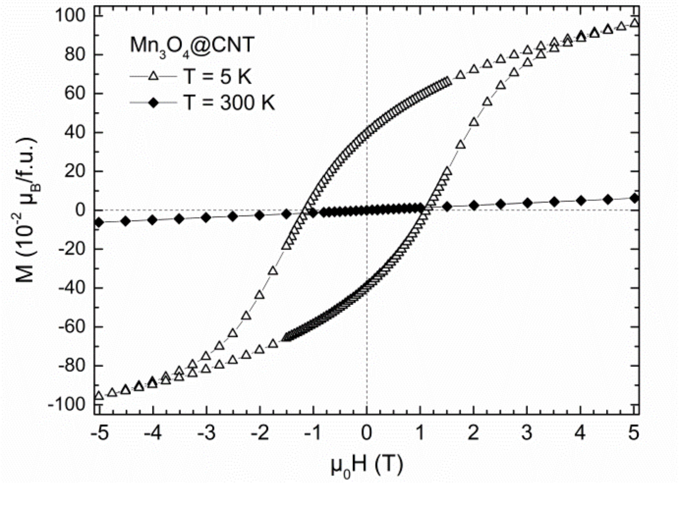

Figure S3. Hysteresis curve of Mn_3O_4@CNT obtained at a temperature of 5 K.
